# A randomized controlled prospective study comparing surgical outcomes of median umbilical ligament lift-up and Veress needle entry techniques in gynecologic laparoscopic surgery

**DOI:** 10.55730/1300-0144.6063

**Published:** 2025-08-05

**Authors:** Gazi GÜNER, Ayşe BACAKSIZ, Can Berk KARABUDAK, Figen EFE ÇAMİLİ, Çisem ERTOK, Sinem ÖZŞAHİN KILIÇ, Zeliha Zeynep SATILMIŞOĞLU, Can TERCAN, Nazlı Aylin VURAL

**Affiliations:** 1 Division of Gynecologic Oncology Surgery, Department of Obstetrics and Gynecology, İstanbul Başakşehir Çam and Sakura City Hospital, İstanbul, Turkiye; 2 Department of Obstetrics and Gynecology, İstanbul Başakşehir Çam and Sakura City Hospital, İstanbul, Turkiye; 3 Department of Obstetrics and Gynecology, Faculty of Medicine, Balıkesir University, Balıkesir, Turkiye; 4 Division of Gynecologic Oncology Surgery, Department of Obstetrics and Gynecology, Bursa Yüksek İhtisas Training and Research Hospital, Bursa, Turkiye; 5 Division of Gynecologic Oncology Surgery, Department of Obstetrics and Gynecology, Faculty of Medicine, Yozgat State Hospital, Yozgat, Turkiye

**Keywords:** Complication, laparoscopic entry, umbilicus lift technique, Veress needle

## Abstract

**Background/aim:**

Laparoscopic surgery has become the preferred minimally invasive technique for both diagnostic and therapeutic procedures in gynecology. Although it is associated with lower overall complication rates compared to open surgery, most serious complications occur during the initial step of abdominal entry. Therefore, developing faster and safer entry methods is critical to improving surgical outcomes. This study aimed to compare the surgical results of the median umbilical ligament lift-up (MULU) technique routinely used in our clinic with the commonly preferred Veress needle (VN) entry technique.

**Materials and methods:**

This randomized, controlled, prospective study included 124 patients: 64 underwent abdominal entry with the MULU technique and 60 with the VN technique. Demographic data such as age, body mass index (BMI), obstetric, surgical history, and menopausal status were recorded. Key perioperative outcomes, including abdominal entry time, number of attempts, insufflation failure, vascular or visceral injury, bleeding at the trocar site, infection, hematoma, and hernia were documented and analyzed.

**Results:**

The VN group had a significantly higher mean age (50.03 years) compared to the MULU group (45.42 years) (p < 0.05). No significant differences were observed in height, weight, or BMI. The MULU technique had a significantly shorter mean entry time compared to VN (71.5 vs. 146.3 s, respectively, p < 0.001). Extraperitoneal insufflation occurred in 6.7% of VN cases and was absent in the MULU group (p = 0.036). Gastric or intestinal injury occurred in 3.3% of VN patients, with none observed in the MULU group. Omental injury was only seen in the MULU group (3.1%). No significant differences were found in vascular injury, bleeding, or prior surgical history.

**Conclusions:**

The MULU technique, based on anatomical guidance via the median umbilical ligament, is a safe and effective method for abdominal entry. It offers faster access and may reduce the risk of major complications, making it a viable alternative to conventional techniques.

## 1. Introduction

Laparoscopic surgery is currently the preferred surgical technique over open surgery due to several advantages, including shorter hospitalization, a quicker return to daily activities, reduced postoperative pain, and decreased intraoperative blood loss [1]. It has become the primary approach for diagnosing and treating gynecological diseases, favored for both benign and malignant conditions involving the uterus, ovaries, and fallopian tubes, as well as for diagnostic evaluations in cases like chronic pelvic pain and infertility [2]. Additionally, laparoscopic methods have lower complication rates of up to 40% compared to conventional open surgery [3]. The initial step of laparoscopic surgery involves inserting surgical instruments through a small abdominal incision to establish pneumoperitoneum. During this step, entry into the abdominal cavity relies primarily on the surgeon’s perception to feel the peritoneal entry or interpret limited visual cues, making it a high-risk maneuver. Although serious complications, such as bowel, bladder, and vascular injuries, occur at a rate of 3–4 per 1000 procedures, more than 50% of these injuries happen during the insertion of the first trocar and may prove fatal if the diagnosis is delayed [4]. Moreover, minor complications like postoperative surgical site infections, subcutaneous emphysema, trocar-site hernias, and insufflation in the extraperitoneal space may also be associated with the technique used for abdominal entry [4]. The increasing use of laparoscopy in patients with a history of prior laparoscopic or open abdominal surgery, as well as in obese or extremely thin patients, has prompted the development of various abdominal entry techniques designed to reduce entry-related complications. While the Veress needle (VN) technique, offering direct trocar entry and access with an optical trocar, and the Hasson technique are among the most widely used methods, many other abdominal entry techniques have also been developed [5]. The development of fast and safe techniques is essential for reducing complications in high-risk patients. The median umbilical ligament is a fibrous remnant of the urachus, a structure located between the allantois and the cloaca during the embryonic period. It extends from the apex of the bladder to the umbilicus on the inner surface of the anterior abdominal wall [6]. At our gynecology clinic, we have developed an easy-to-perform alternative entry method called the median umbilical ligament lift-up (MULU) technique. In our study, we evaluated the results of this new MULU technique against the closed entry VN technique.

## 2. Materials and methods

The abdominal MULU entry technique has been utilized for a brief period in laparoscopic gynecological surgeries at İstanbul Başakşehir Çam and Sakura Research Hospital. This study was designed as a prospective randomized controlled trial and was initiated following approval from the institutional ethics committee in October, 2024. A total of 124 patients aged between 18 and 75 years, all of whom provided written informed consent to participate, underwent elective laparoscopic surgery in the gynecology department for either benign or malignant indications. Exclusion criteria were as follows: history of prior midline incision, diagnosis of a malignancy other than gynecological, chronic corticosteroid use, presence of a preoperative hernia, prior hernia surgery, previous radiotherapy and/or chemotherapy, intraoperative bowel resection and/or anastomosis during the same surgical session, conversion to open surgery, presence of umbilical piercing, patients under the age of 18 years, and patients who declined to participate in the study.

Patients with advanced-stage gynecologic malignancies requiring upper abdominal exploration, bowel resection, or cytoreductive procedures were excluded. Only early-stage cases confined to the uterus or adnexa were included. Diagnoses consisted mainly of early-stage endometrial carcinoma (FIGO grades 1–3), atypical endometrial hyperplasia, and mixed-type carcinomas. Patients with extensive peritoneal disease or bulky adnexal masses were not enrolled.

Participants were randomized using random number tables and divided into 2 groups: those who underwent the VN technique and those who underwent the MULU technique. Demographic characteristics of all participants were recorded, along with their obstetric and surgical histories and menopausal status. The two groups were compared in terms of abdominal entry time, vascular, omental, or visceral organ injuries, and insufflation failures. The laparoscopic entry time was defined as the duration from the initial incision to the visualization of the abdominal cavity and was recorded using a chronometer.

### 2.1. Surgical principles

The surgical procedure was performed under general anesthesia in the dorsal lithotomy position following appropriate cleaning with povidone iodine and sterile covering. As a standard procedure, anticoagulant therapy was discontinued 24 h prior to the surgical intervention, and all patients received 2 g of cefazolin as antibiotic prophylaxis for surgical-site-related infections before the incision. The gastric air was evacuated using a nasogastric or orogastric tube.

#### 2.1.1. VN technique

After making a 5 mm vertical intraumbilical incision, the anterior abdominal wall is raised using towel clamps on both sides. A disposable VN, with a mechanism that has been tested for proper function, is inserted perpendicularly through the incision. After confirming correct placement with the double-click sound and the syringe aspiration-irrigation test, the needle is connected to the CO_2_ insufflation tubing, and insufflation begins at a low flow rate (2 L/min). When the intraabdominal pressure reaches 10 mmHg, a 10 mm trocar is inserted into the abdominal cavity at approximately a perpendicular angle through the incision, and a telescope is introduced to visualize the abdominal cavity. In patients where pneumoperitoneum cannot be established after 3 attempts, VN entry is deemed unsuccessful, and an alternative method is used.

#### 2.1.2. MULU technique

**Figure 1:**
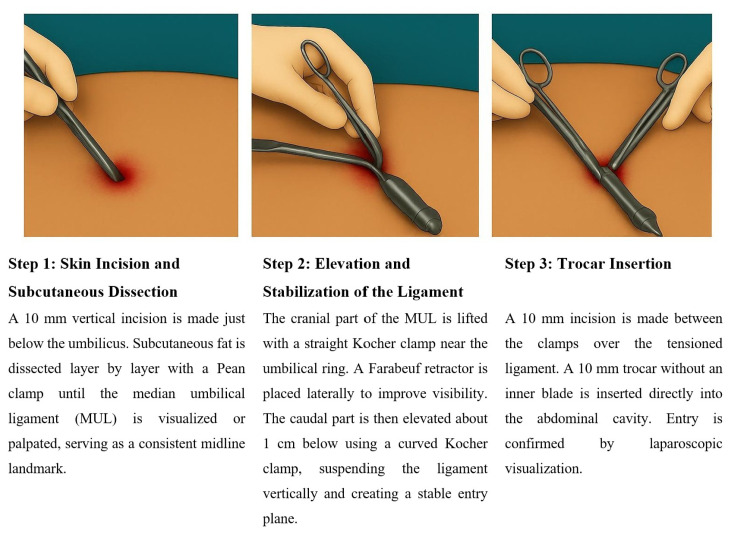
Median umbilical ligament lift-up technique.

After making a 10 mm vertical skin incision below the umbilicus, the subcutaneous fat is dissected using a Pean clamp until the median umbilical ligament was visualized or palpated. The cranial part of the median umbilical ligament, which connects to the umbilical ring, is elevated with a straight Kocher clamp. Next, a Farabeuf retractor is placed on one side of the median umbilical ligament, and the caudal part is lifted with a curved Kocher clamp placed 1 cm below the first clamp. Finally, a 10 mm incision was made between the 2 Kocher clamps. Through this incision, a 10 mm central trocar without an inner blade was inserted into the abdominal cavity, allowing for visualization of the abdominal organs. The key anatomical steps of this procedure are illustrated in Figure 1.

### 2.2. Statistical analysis

A priori, the minimum sample size was calculated as 59 per group based on α error of 0.05, power of 0.99, and effect size d of 0.8 using a 2-tailed t-test in G*Power (version 3.1.9.7). Independent sample Student’s t-test was used to compare the variables. During the analysis, the distributions of the variables were examined, and graphical plots were created for representation. Additionally, in the context of subgroup analyses, one-way analysis of variance (ANOVA) and Tukey’s posthoc tests were conducted. All analyses were performed using Jamovi (version 2.3.21).

## 3. Results

**Figure 2:**
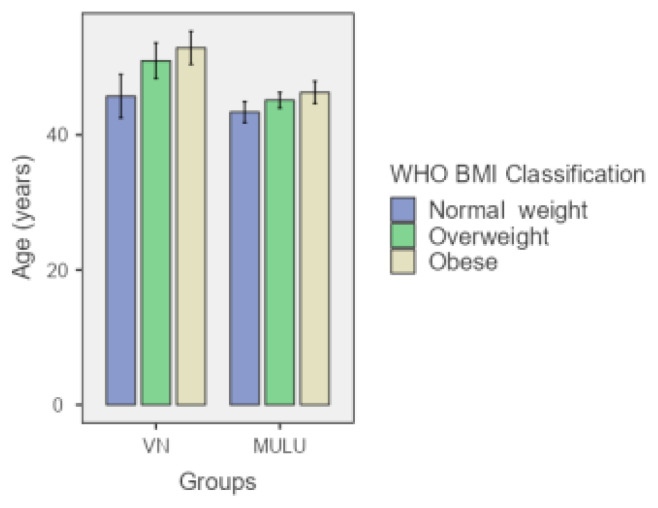
Age distribution across BMI classes in VN and MULU groups. The bars show the mean age and the error bars represent the standard error.

**Figure 3:**
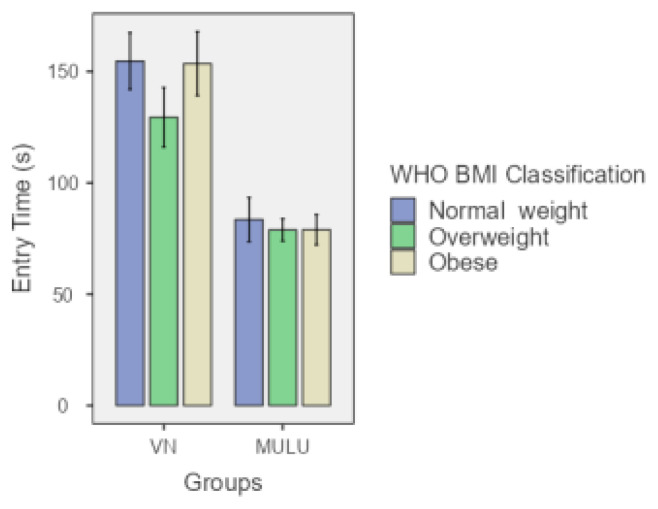
Entry time comparison across BMI classes in VN and MULU. The bars show the mean entry times and the error bars represent the standard error.

**Figure 4:**
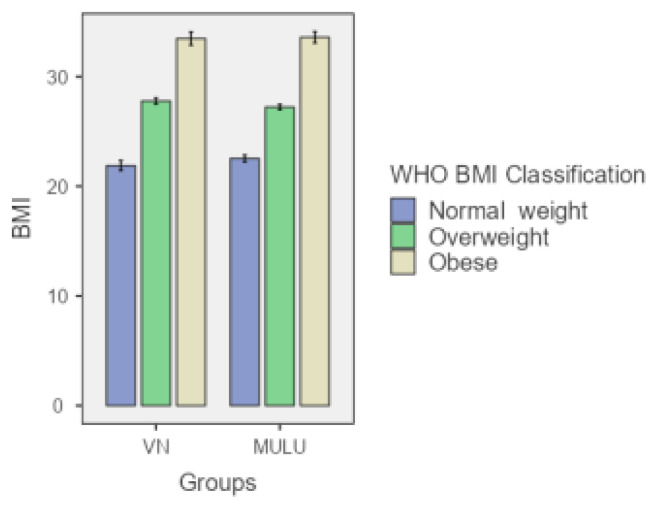
BMI comparison across BMI classes in VN and MULU groups. The bars show the mean BMI and the error bars represent the standard error.

The study included 124 patients, with 60 in the VN group and 64 in the MULU technique group. The mean age was significantly higher in the VN group (50.03 years) than in the MULU group (45.42 years). However, the differences in height, weight, and body mass index (BMI) between the 2 groups were not statistically significant. The mean entry time was significantly longer in the VN group, with an average of 146.32 s versus 71.50 s in the MULU group (p < 0.001) (Table 1). Figures 2–4 illustrate the comparison of patients’ ages, BMIs, and entry time between the groups.

In the VN group, 41.7% of patients had no prior surgical history, compared to 42.4% of the MULU group. Of those with prior surgeries, cesarean section was the most prevalent, occurring at similar rates in both groups: 30.0% for VN and 31.2% for MULU. Other frequently reported previous surgeries included laparoscopic cholecystectomy, noted in 10% of the VN group and 6.2% of the MULU group, as well as appendectomy, seen in 8.3% of VN patients. Additionally, 6.2% of patients in both the VN and MULU groups underwent appendectomy. The distribution of these prior surgeries was comparable between the 2 groups (Table 2).

Extraperitoneal insufflation occurence was significantly higher in the VN group (6.7% of cases), while it was not seen in any case in the MULU group (p = 0.036) (Table 3). Gastric or intestinal injuries were observed in 3.3% of the VN group, with none reported in the MULU group (p = 0.141). In the VN group, 2 patients experienced small bowel serosal injuries. These were identified intraoperatively and successfully repaired laparoscopically without the need for conversion to laparotomy. Postoperative courses were uneventful. Regarding omental injuries, none were reported in the VN group, while 3.1% were observed in the MULU group. The 2 cases of omental injury in the MULU group were minor and did not necessitate conversion to laparotomy or prolong operative time. Contrary to expectations, omental injuries were somewhat more prevalent in the MULU group, but this difference was not statistically significant. Vascular injuries, bleeding at the trocar entry site, and prior surgery history were similar across both groups, with no significant differences. Moreover, complications such as infection, hematoma, and hernia were not observed in either group. These results suggest that both access methods are safe in terms of minor postoperative complications (Table 3).

The World Health Organization classifies individuals by BMI into distinct groups: normal weight (BMI 18–25), overweight (BMI 25–30), and obese (BMI 30–40). A posthoc subgroup analysis was conducted based on this classification. Each BMI subgroup of the MULU group had a statistically significant shorter entry time compared to each BMI subgroup of the VN group. (Table 4).

Among the 124 patients, 65 were diagnosed with malignant or premalignant conditions. The most common was endometrioid adenocarcinoma FIGO grade 2 (23.6%), followed by grade 1 (9.8%), and grade 3 (3.3%). Additionally, 8.1% had atypical endometrial hyperplasia, and 6.4% were diagnosed with mixed-type or serous carcinomas (Table 5).

## 4. Discussion

Our findings indicated that using the MULU technique was significantly faster than with the VN technique. In terms of complications, extraperitoneal insufflation occurred significantly more frequently in the VN group. In the VN group, gastric or bowel injuries were seen in 3.3% of patients, whereas none were reported in the MULU group. Interestingly, against our expectations, omental injury was noted in 3.1% of patients in the MULU group, with a notable history of previous laparoscopic surgery among those affected. Both major and minor vascular injuries, along with trocar-site bleeding, happened at comparable rates in both groups. No instances of hematoma, infection, or hernia were recorded in either group, suggesting that both techniques are safe in terms of postoperative complications.

In laparoscopic surgery, various techniques have been developed to ensure safe and rapid entry into the abdominal cavity. The most commonly used methods include direct trocar insertion, the open (Hasson) technique, and the closed technique using a VN. Numerous studies in the literature have investigated the advantages and comparative effectiveness of these techniques. In a randomized controlled study including 135 obese women comparing the direct trocar technique and the VN entry technique, the abdominal entry time was found to be shorter in the direct trocar group; however, there was no significant difference in the number of attempts for successful entry, major complications, or minor complications [7]. In a systematic review that included 16 randomized controlled trials and 5 observational studies, no significant difference was found in major complications during the entry maneuver when comparing the VN group with the direct trocar entry group; however, the rate of minor complications was lower, and the number of unsuccessful entries and attempts was reduced in the direct trocar group [8]. In a prospective study involving 453 patients, 3 techniques were compared: direct trocar, VN, and open technique. A total of 3 major complications were reported in the direct trocar group, including 1 colon perforation and 2 iliac artery injuries, while 1 major complication was reported in the VN group, which included 1 iliac artery injury. There were no major complications in the open technique group. The frequency of minor complications was ranked as follows: open technique, VN, and direct trocar, respectively. Gas leakage was more common in the open technique [9]. Kumar et al. [10] found that there was no difference between the open and closed techniques in terms of minor and major complications in a prospective study; however, the time to enter the peritoneal cavity and the time required for umbilical port closure were shorter with the open technique. In a prospective study of 200 patients, open and closed entry techniques were compared. No major vascular or visceral injuries were observed in the open entry technique, and it was considered superior to the closed technique in terms of complications [11]. In a randomized controlled study involving 400 patients, the direct trocar technique was compared with the VN technique. A higher incidence of extraperitoneal insufflation was observed with the VN. Omental injury was also more common in the VN group, and the rate of port-site infection was higher in this group as well [12].

Although the open technique is considered the safest method for abdominal entry, it has disadvantages in obese patients due to the difficulty of lifting the thick fascia, increased subcutaneous emphysema, and a higher risk of hernia development. The VN technique has become the preferred method for many gynecologists as a classic entry technique [13,14]. However, vascular and visceral organ injuries are observed more frequently with this technique compared to the open method, highlighting the need for the development of specialized entry techniques to avoid such complications. Due to its weak structure and ease of entry, the umbilicus is preferred for VN insertion. It is essential to understand the embryological development of the umbilicus. The umbilical ring is the anatomical region where the ligamentum teres hepatis, medial umbilical ligaments, and median umbilical ligament converge, representing the weakest area of the abdominal wall. The medial umbilical ligaments form the sides of the ring, while the median ligament, located in the midline, extends from above the bladder to the umbilical ring. The ligamentum teres hepatis originates from the anterior surface of the liver and extends toward the umbilical ring [15].

Based on these anatomical landmarks of the umbilical ring, in 2023, Afsar et al. [16] described a novel technique for abdominal entry. This technique, named the “teres lift-up” method, was evaluated for its safety in both obese and normal-weight women when compared to the traditional VN entry. Building upon this anatomical knowledge, we developed our own technique for abdominal entry. In this prospective study, we present our clinical experience and evaluate the outcomes associated with this method. The new technique we used provided advantages over the VN technique, such as faster entry into the abdominal cavity, fewer visceral injuries, and less extraperitoneal insufflation.

## 5. Conclusion

In our prospective study, we showed that the MULU technique, which involves entering the abdomen by elevating the median umbilical ligament, is a fast and safe alternative for preventing vascular and visceral injuries. The technique is based on anatomical principles and may facilitate the definition, teaching, and wider adoption of the technique.

## Figures and Tables

**Table 1:** Demographic and descriptive statistics.

Characteristic	VN (N = 60)	MULU (N = 64)	p value
Age (Mean ± SD)	50.0 ± 12.5	45.4 ± 8.0	**0.015**
Weight (Mean ± SD)	74.9 ± 13.2	73.2 ± 12.0	0.446
Height (Mean ± SD)	163.9 ± 6.91	164.3 ± 5.65	0.748
BMI (Mean ± SD)	28.0 ± 5.26	27.2 ± 4.77	0.378
Entry time (s, Mean ± SD)	146.3 ± 61.0	71.5 ± 19.1	**<0.001**
Menopause status n (%)	34 (56.7%)	18 (28.1%)	**<0.001**

**Abbreviations:** BMI: Body Mass Index, MULU: Median Umbilical Ligament Lift-Up, SD: Standard Deviation, VN: Veress Needle

**Table 2:** History of previous surgeries.

Previous Surgery	VN (N = 60)	MULU (N = 64)	Total (N = 154)	p value
None n (%)	25 (41.7%)	27 (42.2%)	52 (41.9%)	0.410
LS BTL n (%)	1 (1.7%)	0 (0%)	1 (0.8%)	
C-section n (%)	18 (30%)	20 (31.2%)	38 (30.6%)	
LT appendectomy n (%)	5 (8.3%)	4 (6.2%)	9 (5.8%)	
LS cholecystectomy n (%)	6 (10%)	4 (6.2%)	10 (8.1%)	
LS salpingectomy n (%)	1 (1.7%)	0 (0%)	1 (0.8%)	
LT Burch n (%)	1 (1.7%)	0 (0%)	1 (0.8%)	
LT myomectomy n (%)	1 (1.7%)	0 (0.0%)	1 (0.8%)	
LT cystectomy n (%)	1 (1.7%)	0 (0.0%)	1 (0.8%)	
LS detorsion n (%)	0 (0%)	1 (1.6%)	1 (0.8%)	
LS cystectomy n (%)	0 (0%)	3 (4.7%)	3 (1.9%)	
LS USO n (%)	0 (0%)	1 (1.6%)	1 (0.8%)	
LS isthmocele n (%)	0 (0%)	1 (1.6%)	1 (0.8%)	
LS ectopic n (%)	0 (0%)	1 (1.6%)	1 (0.8%)	
TAH + BSO n (%)	0 (0%)	2 (3.1%)	2 (1.6%)	

**Abbreviations:** BTL: Bilateral Tubal Ligation, LS: Laparoscopic, LT: Laparotomic, MULU: Median Umbilical Ligament Lift-Up, TAH + BSO: Total Abdominal Hysterectomy with Bilateral Salpingo-Oophorectomy, USO: Unilateral Salpingo-Oophorectomy, VN: Veress Needle.

**Table 3:** Complications in VN and MULU groups.

Complication	VN (N = 60)	MULU (N = 64)	p value
Extraperitoneal insufflation n (%)	4 (6.7%)	0 (0%)	**0.036**
Vascular damage n (%)	6 (10.0%)	6 (9.4%)	0.906
Omental injury n (%)	0 (0%)	2 (3.1%)	0.167
Gastric or intestinal injury n (%)	2 (3.3%)	0 (0%)	0.141
Trocar site bleeding n (%)	1 (1.7%)	2 (3.1%)	0.597
Infection n (%)	0 (0%)	0 (0%)	-
Hematoma n (%)	0 (0%)	0 (0%)	-
Hernia n (%)	0 (0%)	0 (0%)	-

**Abbreviations:** MULU: Median Umbilical Ligament Lift-Up, VN: Veress Needle

**Table 4:** Posthoc subgroup analysis based on BMI class.

Groups	BMI Classification (WHO)	Compared With	BMI Classification (WHO)	Mean Difference	SE	df	t	p value (Tukey)	Cohen’s d
Veress Needle	Overweight	Veress Needle	Obese	−24.12	13.92	118.00	−1.73	0.514	−0.54
		Veress Needle	Normal weight	−25.26	14.42	118.00	−1.75	0.501	−0.57
		MULU	Overweight	56.34	13.92	118.00	4.05	**< 0.001**	1.27
		MULU	Obese	52.16	14.42	118.00	3.62	0.006	1.17
		MULU	Normal weight	64.34	13.78	118.00	4.67	**< 0.001**	2.01
	Obese	Veress Needle	Normal weight	−1.15	13.92	118.00	−0.08	1.000	−0.03
		MULU	Overweight	80.45	13.40	118.00	6.00	**< 0.001**	−1.81
		MULU	Obese	76.28	13.40	118.00	5.69	**< 0.001**	–1.73
		MULU	Normal weight	88.46	13.26	118.00	6.67	**< 0.001**	1.99
	Normal weight	MULU	Overweight	81.60	13.92	118.00	5.86	**< 0.001**	−1.84
		MULU	Obese	77.42	14.42	118.00	5.37	**< 0.001**	−1.72
		MULU	Normal weight	89.61	13.78	118.00	6.50	**< 0.001**	2.02
MULU	Overweight	MULU	Obese	−4.18	13.92	118.00	−0.30	1.000	−0.09
		MULU	Normal weight	−8.03	13.26	118.00	−0.60	0.991	−0.18
	Obese	MULU	Normal weight	12.19	13.78	118.00	0.88	0.950	0.27

Note: Comparisons are based on estimated marginal means

**Abbreviations:** Cohen’s d: Cohen’s d effect size, df: Degrees of Freedom, MULU: Median Umbilical Ligament Lift-Up, p (Tukey): p-value from Tukey’s posthoc test, SE: Standard Error, t: t-value

**Table 5:** Final pathology results (n = 124).

Diagnosis	n	%
Benign pathology	59	48.0
Endometrioid adenocarcinoma, FIGO Grade 2	29	23.6
Endometrioid adenocarcinoma, FIGO Grade 1	12	9.8
Atypical endometrial glandular hyperplasia	10	8.1
Endometrioid adenocarcinoma, FIGO Grade 3	4	3.3
Mixed type carcinoma (endometrioid + serous carcinoma)	3	2.4
Serous carcinoma	2	1.6
Mixed type carcinoma (endometrioid + clear cell carcinoma)	2	1.6
Endometrioid adenocarcinoma (endometrium + ovary involvement)	1	0.8
Mixed type carcinoma (carcinosarcoma)	1	0.8
Endometrioid adenocarcinoma (with clear cell changes)	1	0.8

**Abbreviations:** FIGO: International Federation of Gynecology and Obstetrics
